# Effect of Tranexamic Acid against Staphylococcus spp. and Cutibacterium acnes Associated with Peri-Implant Infection: Results from an *In Vitro* Study

**DOI:** 10.1128/spectrum.01612-21

**Published:** 2022-02-16

**Authors:** Antonio Benjumea, Marta Díaz-Navarro, Rama Hafian, Mar Sánchez-Somolinos, Javier Vaquero, Francisco Chana, Patricia Muñoz, María Guembe

**Affiliations:** a Department of Orthopaedic Surgery and Traumatology, Hospital General Universitario Gregorio Marañón, Madrid, Spain; b Department of Clinical Microbiology and Infectious Diseases, Hospital General Universitario Gregorio Marañón, Madrid, Spain; c Instituto de Investigación Sanitaria Gregorio Marañón, Madrid, Spain; d School of Biology, Universidad Complutense de Madrid, Madrid, Spain; e CIBER Enfermedades Respiratorias-CIBERES (CB06/06/0058), Madrid, Spain; f School of Medicine, Universidad Complutense de Madrid, Madrid, Spain; Weizmann Institute of Science

**Keywords:** tranexamic acid, bacterial growth, biofilm, antimicrobial effect, *in vitro* model

## Abstract

Tranexamic acid (TXA) is extensively used in orthopedic surgery and traumatology as an antifibrinolytic agent to control intra- and postoperative bleeding and, therefore, indirectly, to reduce postsurgery infection rates. The hypothesis of an additional antibiotic effect against microorganisms associated with periprosthetic joint infection needs to be further evaluated. We aimed to assess whether TXA could reduce bacterial growth using an *in vitro* model. ATCC and clinical strains of staphylococci and Cutibacterium acnes were tested against TXA in both planktonic and sessile forms. We recorded the percent reduction in the following variables: log CFU/mL by microbiological culture, percentage of live cells by confocal laser scanning microscopy, and, additionally in sessile cells, metabolic activity by the 2,3-bis-(2-methoxy-4-nitro-5-sulfophenyl)-2H-tetrazolium-5-carboxanilide salt (XTT) assay. Variables were compared between groups using the Kruskal-Wallis test, and the results were reported as median (interquartile range [IQR]). Statistical significance was set at a *P* value of <0.05. Clinical significance was defined as a reduction of ≥25%. TXA at 50 mg/mL led to a slight reduction in CFU counts (4.5%). However, it was at 10 mg/mL that the reduction reached 27.2% and 33.0% for log CFU/mL counts and percentage of live cells, respectively. TXA was not efficacious for reducing preformed 24-h mature staphylococci and 48-h mature *C. acnes* biofilms, regardless of its concentration. TXA did not exert an antimicrobial effect against bacterial biofilms. However, when bacteria were in the planktonic form, it led to a clinically and statistically significant reduction in bacterial growth at 10 mg/mL.

**IMPORTANCE** The possible use of TXA as an antibiotic agent in addition to its antifibrinolytic effect may play an important role in the prevention of prosthetic joint infection.

## INTRODUCTION

Tranexamic acid (TXA) is increasingly used as a hemostatic agent in daily clinical practice. Numerous studies support the use of this drug for the control of perioperative bleeding in several elective surgical or emergency procedures, for example, in patients with multiple trauma (1, 2). Use of TXA in surgical interventions, specifically in orthopedic surgery and traumatology, has improved the postoperative management of patients undergoing both elective and emergency surgery (3, 4).

TXA acts as an antifibrinolytic agent that binds to plasminogen. Once it is activated to plasmin, it degrades fibrin and thus enables blood clotting (3, 5). Moreover, its use has not significantly increased complications in the healthy population (6).

Periprosthetic joint infection (PJI) accounts for 25.2% and 14.8% of revisions after knee and hip arthroplasty (7). These infections are caused by bacterial biofilm that adheres to the prosthesis and resists host defenses and antibiotics. Therefore, preventive strategies should be implemented to reduce PJI at the time of surgery (8).

Several clinical studies have demonstrated that TXA significantly reduced wound complications. It also seems to decrease infectious complication rates. Some authors point out that by reducing local hematoma, TXA could indirectly reduce infection rates (9–13). Whether, in addition to its antifibrinolytic action, TXA has a potential antibiotic effect, as demonstrated for hyaluronic acid (7), needs to be clarified. Zhang et al. recently observed in an *in vivo* model that continuous local injection of TXA could promote biofilm formation of Staphylococcus aureus, whereas single local injection had no influence on bacterial load (14).

Therefore, we aimed to assess whether TXA could have an additional direct antibiotic effect when administered as an intraarticular injection against the growth of Staphylococcus spp. and Cutibacterium acnes under experimental conditions.

## RESULTS

### Planktonic model.

**FIG 1:**
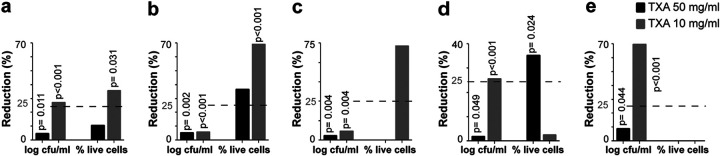
Median (IQR) percent reduction in log CFU/mL and percentage of live cells in the planktonic model. (a) Overall (all microorganisms together). (b) MSSA. (c) MRSA. (d) S. epidermidis. (e) *C. acnes*.

We found statistically and clinically significant differences between median percent reduction rates in bacterial counts and cell viability for 10 mg/mL TXA (27.2% and 33.0%, respectively). In addition, statistical significance was achieved for 50 mg/mL TXA in bacterial counts (4.9%; *P* = 0.011) (Fig. 1a). Details of the median (interquartile range [IQR]) log CFU/mL and percentage of live cells are summarized in Table 1.

**TABLE 1:** Median (IQR) log CFU/mL and percentage of live cells in the planktonic model according to each group of treatment

Microorganism^*a*^	Treatment^*a*^	Median (IQR^*a*^) log CFU/mL	*P* value^*b*^	Median (IQR) % live cells	*P* value^*b*^
Overall	+C	8.98 (6.40–9.20)		92.7 (5.40–100)	
	TXA 50 mg/mL	8.58 (5.30–9.20)	**0.011**	90 (0.0–100)	0.539
	TXA 10 mg/mL	7.69 (0.00–9.00)	**<0.001**	69.8 (0.0–100)	**0.031**
MSSA	+C	8.92 (8.48–9.11)		91.7 (22.2–98.7)	
	TXA 50 mg/mL	8.47 (8.00–8.60)	**0.002**	79.7 (2.0–100)	0.083
	TXA 10 mg/mL	8.35 (7.88–8.95)	**<0.001**	24.4 (0.0–76.6)	**<0.001**
MRSA	+C	9.10 (9.00–9.15)		72.1 (5.4–100)	
	TXA 50 mg/mL	8.71 (8.70–8.86)	**0.004**	19.2 (0.0–95.7)	0.199
	TXA 10 mg/mL	8.56 (7.78–8.78)	**0.004**	12.0 (1.4–71.4)	0.054
S. epidermidis	+C	9.11 (9.04–9.18)		98.2 (71.7–99.1)	
	TXA 50 mg/mL	9.00 (8.52–9.18)	**0.049**	79.3 (1.2–99.4)	**0.024**
	TXA 10 mg/mL	7.36 (3.18–9.00)	**<0.001**	76.1 (0.0–100)	0.272
*C. acnes*	+C	6.65 (6.40–7.20)		89.5 (60.7–100)	
	TXA 50 mg/mL	6.00 (5.30–7.08)	**0.044**	100 (92.9–100)	<0.001
	TXA 10 mg/mL	1.65 (0.00–5.11)	**<0.001**	100 (92.3–100)	0.004

a IQR, interquartile range; +C, positive control; TXA, tranexamic acid; MSSA, methicillin-susceptible Staphylococcus aureus; MRSA, methicillin-resistant Staphylococcus aureus.

b *P* values in bold represent statistical significance for the TXA reduction effect.

The highest values of median percent reduction in cell viability were observed for S. aureus: 69.3% and 73.0% for methicillin-susceptible Staphylococcus aureus (MSSA) and methicillin-resistant Staphylococcus aureus (MRSA), respectively (Fig. 1b and c). This corresponds to a median percentage of live cells in the positive controls and 10 mg/mL TXA of 91.7% versus 24.4% (*P* < 0.001) and 72.1% versus 12.0% (*P* = 0.054) for MSSA and MRSA, respectively (Table 1).

In the case of S. epidermidis, although differences for 50 mg/mL TXA reached statistical significance for both bacterial counts and cell viability (median log CFU/mL of 9.00 versus 9.11, *P* = 0.049; median percentage of live cells: 79.3% versus 98.2%, *P* = 0.024), clinical significance was only achieved for cell viability (32.4%) (Table 1; Fig. 1d). For 10 mg/mL TXA, statistical and clinical significance were only reached for bacterial counts (25.6% reduction, *P* < 0.001) (Fig. 1d).

In the case of *C. acnes*, a statistically significant reduction in bacterial counts was observed for both 50 mg/mL TXA and 10 mg/mL TXA (8.7% and 69.9%, respectively). The median (IQR) log CFU/mL values were, respectively, 6.00 (5.30 to 7.08) and 1.65 (0.00 to 5.11) versus 6.65 (6.40 to 7.20) for the positive controls (*P* = 0.044 and *P* < 0.001) (Table 1; Fig. 1e).

### Biofilm model.

**FIG 2:**
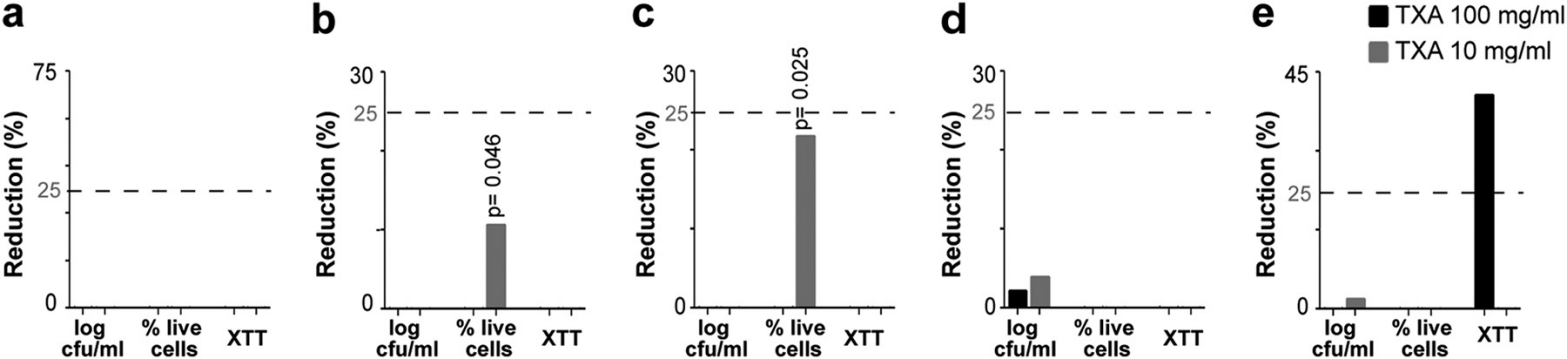
Median (IQR) percent reduction in log CFU/mL, percentage of live cells, and XTT absorbance in the biofilm model. (a) Overall (all microorganisms together). (b) MSSA. (c) MRSA. (d) S. epidermidis. (e) *C. acnes*.

Treatment of preformed 24-h mature biofilms did not reveal statistically or clinically significant results with TXA at 50 mg/mL or at 10 mg/mL (Table 2; Fig. 2a).

**TABLE 2:** Median (IQR) log CFU/mL, percentage of live cells, and absorbance of XTT in the biofilm model according to each group of treatment

Microorganism^*a*^	Treatment^*a*^	Median (IQR^*a*^) log CFU/mL	*P* value^*b*^	Median (IQR) % live cells	*P* value^*b*^	Median (IQR) absorbance of XTT	*P* value^*b*^
Overall	+C	6.88 (3.60–8.20)		79.5 (4.5–100)		0.226 (0.009–0.967)	
	TXA 100 mg/mL	7.43 (3.50–8.40)	0.130	78.4 (25.0–100)	0.512	0.271 (0.000–1.805)	0.260
	TXA 10 mg/mL	6.95 (3.70–8.20)	0.862	76.5 (4.9–100)	0.472	0.200 (0.000–0.891)	0.907
MSSA	+C	7.15 (5.30–7.88)		89.1 (77.3–100)		0.331 (0.223–0.962)	
	TXA 100 mg/mL	7.60 (4.78–8.40)	0.172	96.7 (72.3–100)	0.206	0.276 (0.189–1.805)	0.954
	TXA 10 mg/mL	7.4 (5.78–8.2)	0.174	78.7 (64.7–100)	**0.046**	0.287 (0.183–0.773)	0.356
MRSA	+C	7.04 (6.88–7.11)		84.7 (70.4–90.9)		0.664 (0.409–0.967)	
	TXA 100 mg/mL	7.63 (7.26–8.00)	0.003	88.2 (65.6–93.9)	0.630	0.604 (0.469–0.857)	0.873
	TXA 10 mg/mL	7.10 (7.08–7.08)	0.098	62.1 (57.0–87.0)	**0.025**	0.742 (0.463–0.891)	1
S. epidermidis	+C	7.45 (5.95–8.20)		66.8 (8.1–88.7)		0.175 (0.059–0.387)	
	TXA 100 mg/mL	7.37 (5.78–8.11)	0.401	77.3 (52.6–91.1)	0.149	0.191 (0.165–0.397)	0.371
	TXA 10 mg/mL	6.87 (5.48–8.18)	0.386	80.3 (68.9–94.1)	0.073	0.189 (0.044–0.354)	0.544
*C. acnes*	+C	4.95 (3.60–3.48)		74.7 (10.0–91.9)		0.208 (0.024–0.453)	
	TXA 100 mg/mL	4.91 (3.48–8.00)	1	60.5 (25.0–71.4)	1.333	0.070 (0–0.256)	0.337^*c*^
	TXA 10 mg/mL	4.75 (3.74–7.26)	0.544	75.6 (20.7–100)	0.488	0.171 (0–0.330)	0.688^*c*^

a IQR, interquartile range; +C, positive control; TXA, tranexamic acid; MSSA, methicillin-susceptible Staphylococcus aureus; MRSA, methicillin-resistant Staphylococcus aureus.

b *P* values in bold represent statistical significance for the TXA reduction effect.

c Tested in only two species.

When the analysis was performed by species, we detected no effect on S. epidermidis (Table 2; Fig. 2d). In contrast, statistical significance was achieved for reduced cell viability in MSSA and MRSA treated with 10 mg/mL TXA with respect to the positive control (median [IQR] percentage of live cells, 78.7 [64.7 to 100] versus 89.1 [77.3 to 100], *P* = 0.046, and 62.1 [57.0 to 87.0] versus 84.7 [70.4 to 90.9], *P* = 0.025, respectively) (Table 2; Fig. 2b and c).

In addition, the reduction in metabolic activity for *C. acnes* biofilms treated with 100 mg/mL TXA (40.6%) was significant (Table 2; Fig. 2e).

## DISCUSSION

We demonstrated that TXA exerted an antibiotic effect against planktonic bacteria, mostly in S. aureus and *C. acnes*. In contrast, when a mature biofilm was already established, no reduction was possible.

Administration of TXA significantly reduced postsurgery infection rates, as it controls bleeding through its antifibrinolytic effect (9–13). Infection rates were significantly lower among nondiabetic patients who received TXA during surgery (*P* = 0.003) (5). After cardiac surgery, TXA was shown to modulate myeloid and lymphoid cells (causing significant changes in innate immune cells and T cells) and plasma cytokines (reducing proinflammatory cytokine levels), which participate in the immune response (5).

We hypothesized that the reduction in PJI rates could be due either to (i) the indirect effect by immunomodulatory mechanisms and by the reduction in the frequency of the postoperative hematoma, which in itself is a medium that favors bacterial growth, or (ii) the direct effect of the drug itself on bacterial growth, as occurs with hyaluronic acid (2, 5, 7–12). We based our hypothesis on the fact that, as lysine analogs inhibited survival of and colonization by many bacteria and given that TXA is a lysine analog that reversibly binds to plasminogen, it would therefore have some antibiotic effect (2, 15).

The dose of TXA used by most of the authors in the literature is between 1 and 3 g. As the joint volume of one knee is estimated at 131 mL (±53 mL), the intraarticular TXA dilution would be approximately 10 to 30 mg/mL. Therefore, we consider that, as the surface distribution of TXA in the joint that underwent surgery is approximately 65 cm^2^ (16, 17), and 3 g (30 mL) of TXA is used in our institution (46 mg/cm^2^, represented using 100 mg/mL TXA in a 0.32-cm^2^ well), the concentration and the volume finally reached and absorbed in the joint can be reduced. In addition, other authors administered doses of 30 mg/mL (3, 18). Therefore, we decided to test the effect of TXA at 10 mg/mL and also 50 mg/mL, despite that it was slightly above that described to extend the doses, and see whether it has a positive or negative effect as in previous studies (14). However, we do not know whether single or continuous local doses could affect our results as observed in the study of Zhang et al., in which while single doses did not affect microbial growth, continuous doses did (14).

Even though we used preformed biofilms, to which bacteria already adhere, TXA did not show any efficacy at any concentration; we found statistically and clinically significant differences between 10 mg/mL TXA-treated planktonic bacteria and untreated bacteria, both in terms of median CFU counts and live cells (7.69 versus 8.98 [27.2% reduction], *P* < 0.001, and 69.8% versus 92.7% [33.0% reduction], *P* = 0.031, respectively).

Regarding discrepancies observed between median percent reduction rates according to log CFU/mL or viable cells, we consider that it could be due to the phenomenon of “viable but not culturable cells,” which has also been observed in other research areas (19). The significance of this phenomenon is not yet clear, although it is thought that perhaps the cells that are still alive in confocal laser scanning microscopy (CLSM) are really in a predeath phase and, therefore, are not recovered in culture (20).

Regarding the selection of the strains, as most common isolated organisms in PJI are staphylococci followed by anaerobes, we designed our *in vitro* model using ATCC and clinical strains of these microorganisms and found that the effect differed according to genus and species (7). Moreover, differences were also observed according to the variables and the TXA concentration tested. In particular, in the planktonic model, S. aureus (both MSSA and MRSA) and *C. acnes* almost reached a 70% reduction in cell viability and bacterial counts with 10 mg/mL TXA, respectively. In contrast, in S. epidermidis, the percent reduction in bacterial counts and cell viability was 25.6% and 32.4% for 10 mg/mL TXA and 50 mg/mL TXA, respectively. In the biofilm model, while no effect was observed when all microorganisms were assessed together, differences were observed depending on the species, the variable measured, and the concentration of TXA tested. Specifically, 100 mg/mL TXA led to a 40.6% reduction in metabolic activity for *C. acnes*, and 10 mg/mL TXA led to a 10.6% and 21.8% reduction in cell viability for MSSA and MRSA, respectively. Regarding this aspect, it is difficult to compare our results with that of Zhang et al., as they only tested a single strain of S. aureus (14).

As for the differences in results depending on the TXA concentration used, we observed that percent reductions were generally better when the concentration of TXA was lower. Therefore, it would be interesting to test in future studies the effect of TXA at rising concentrations to assess whether a paradoxical effect could exist, as has been demonstrated both with azoles and *Candida* spp. and with ampicillin and Gram-positive cocci (21–23). Our findings did not correlate to those shown in the *in vivo* study of Zhang et al., in which TXA at 50 mg/mL seemed to inhibit S. aureus growth (14).

The main limitation of the study is that our data cannot be extrapolated to other species and that our *in vitro* biofilm model was static and based only on the metabolic activity assay. In addition, no growth curves and time-kill kinetic assays were performed.

To date, this is the first *in vitro* study to assess whether the local use of TXA could have a direct effect on the growth of Gram-positive cocci. Perhaps this drug acts by inhibiting bacterial growth in a similar way to hyaluronic acid, thus helping to prevent PJI in patients in whom it has been administered (7). Further studies are needed to evaluate its activity as an adjuvant to antibiotics and to validate these results with *in vivo* models.

### Conclusion.

TXA at 10 mg/mL significantly reduced bacterial growth in an *in vitro* planktonic model, whereas no antimicrobial effect was observed in sessile cells. Further research in this area would help to confirm our results.

## MATERIALS AND METHODS

The study was carried out at Hospital General Universitario Gregorio Marañón, Madrid, Spain. We selected 16 strains: 4 ATCC strains (methicillin-susceptible Staphylococcus aureus [MSSA; ATCC 29213], methicillin-resistant Staphylococcus aureus [MRSA; ATCC 43300], methicillin-resistant Staphylococcus epidermidis [MRSE; ATCC 35984], and Cutibacterium acnes [ATCC 11827]) and 3 clinical strains of each species (except for MRSA, of which we only had 1) isolated from patients with PJI that had previously proven to be high biofilm producers by the crystal violet and 2,3-bis-(2-methoxy-4-nitro-5-sulfophenyl)-2H-tetrazolium-5-carboxanilide salt (XTT) assays.

We tested TXA at concentrations of 100 mg/mL (commercial preparation) and 50 mg/mL and 10 mg/mL in sterile water (the latter representing the concentration reached in daily clinical practice). Solutions were kept at 2 to 4°C until use. Positive controls were treated with sterile water.

All the experiments were performed in triplicate.

### Planktonic model.

The wells of a 96-well plate were inoculated with 100 μL of a suspension of 10^5^ CFU/mL of each microorganism in Müller-Hinton broth and treated with either 100 μL of the corresponding TXA concentration (100 mg/mL [final concentration of 50 mg/mL, as total volume is 200 μL and concentration is diluted 1:2] and 20 mg/mL [final concentration of 10 mg/mL]) or 100 μL of sterile water (positive controls). Plates were incubated at 37°C for 24 h (48 h in anaerobiosis for *C. acnes*).

**(i) Quantification of bacterial counts (log CFU/mL).** Serial dilutions of the content of the wells were performed, and 100 μL was cultured on blood agar plates (Brucella agar plate for *C. acnes*). Plates were incubated at 37°C for 24 h (48 h in anaerobiosis for *C. acnes*). CFU/plate values were counted, and CFU/mL values were calculated.

The percent reduction in CFU/mL was calculated using equation 1. Values were expressed on a logarithmic scale.

(1) $$\text{\% }\frac{\text{CFU}}{\text{mL}}\text{reduction}=\left[1-\left(\frac{\frac{\text{CFU}}{\text{mL}}\text{ treated strain}}{\frac{\text{CFU}}{\text{mL}}\text{ positive control}}\right)\right]\;\times 100$$

**(ii) Quantification of cell viability.** Aliquots (2 μL) of the content of the wells were mounted on coverslips and stained with 2 μL of BacLight (composed of SYTO 9, which emits green fluorescence for live cells, and propidium iodide, which emits red fluorescence for dead cells). The images were obtained using inverted confocal laser scanning microscopy (CLSM). The ratio of live-to-dead (%) cells was calculated using FIJI software.

The percent reduction in live cells was calculated using equation 2.

(2) $$\text{\% of viability reduction}=\left[1-\left(\frac{\text{\% live cells treated strain}}{\text{\% live cells positive control}}\right)\right]\times \;100$$

### Biofilm model.

Biofilm was formed as described by (24), with some modifications. Briefly, colonies isolated from blood agar culture (Brucella for *C. acnes*) of each strain were inoculated into Falcon tubes with 20 mL of culture broth (tryptic soy broth [TSB] for MSSA and MRSA, TSB supplemented with 1% glucose for MRSE, and brain heart infusion with 1% glucose for *C. acnes*). The strains inoculated into Falcon tubes were incubated at 37°C in an orbital shaker (150 rpm) for 24 h (48 h in anaerobiosis for *C. acnes*). The inoculum was then washed in three centrifuge cycles, resuspended with phosphate-buffered saline (PBS), and adjusted to 0.5 McFarland turbidity (10^8^ CFU/mL). Aliquots of 100 μL were inoculated into 96-well plates, and plates were incubated for 24 h (48 h in anaerobiosis for *C. acnes*) for quantification of metabolic activity and bacterial counts (CFU/mL). After biofilm formation, plates were washed with PBS to eliminate nonadherent cells and were treated with 100 μL of TXA (100 mg/mL and 10 mg/mL) or sterile water (positive controls). Each strain was tested in triplicate, and negative controls were included.

**(i) Quantification of bacterial counts (log CFU/mL).** The procedure was the same as in the planktonic model, with a previous step in which wells were washed with PBS to eliminate nonadherent cells, and their content was scraped into 100 μL of PBS.

**(ii) Quantification of cell viability.** The procedure was the same as in the planktonic model, with a previous step in which wells were washed with PBS to eliminate nonadherent cells, and their content was scraped into 100 μL of PBS.

**(iii) Quantification of metabolic activity.** Wells were washed with PBS to eliminate nonadherent cells, and 100 μL of XTT/menadione solution was added to each well. Plates were incubated at 37°C for 2 h (in anaerobiosis for *C. acnes*) protected from light. Absorbance was then measured at 492 nm in a spectrophotometer.

Data were expressed as the percent reduction in metabolic activity using equation 3.

(3) $$\text{\% of metabolic reduction}=\left[1-\left(\frac{\text{Abs492 treated strain}}{\text{Abs492 positive control}}\right)\right]\times \;100$$

### Statistical and clinical analysis.

Quantitative variables are expressed as median and interquartile range (IQR). We used parametric methods (*t* test or analysis of variance [ANOVA]) or nonparametric methods (median test). Linear or logistic regression models were fitted in cases of asymmetry.

Statistical significance was set at a *P* value of <0.05 for all the tests. The statistical analysis was performed using IBM SPSS Statistics for Windows, version 21.0 (IBM Corp., Armonk, New York, USA).

We also considered a reduction >25% to be clinically significant. We arbitrarily chose a 25% cutoff because we considered it a reasonable value below which the clinical impact would not be significant, as used in other studies of our group (25).
